# Effects of Charge Trapping on Memory Characteristics for HfO_2_-Based Ferroelectric Field Effect Transistors

**DOI:** 10.3390/nano13040638

**Published:** 2023-02-06

**Authors:** Jianjian Wang, Jinshun Bi, Yannan Xu, Gang Niu, Mengxin Liu, Viktor Stempitsky

**Affiliations:** 1 Institute of Microelectronics, Chinese Academy of Sciences, Beijing 100029, China; 2 School of Microelectronics, University of Chinese Academy of Sciences, Beijing 100049, China; 3 School of Electronic Science, Xi’an Jiaotong University, Xi’an 710049, China; 4 Beijing Zhongke New Micro Technology Department Co., Ltd., Beijing 100029, China; 5 Department of Microelectronics, Belarusian State University of Informatics and Radioelectronics, 220015 Minsk, Belarus

**Keywords:** HfO_2_-based FeFET, charge trapping, ferroelectric switching, memory window, pulse amplitude, pulse width, conductance modulation, TCAD

## Abstract

A full understanding of the impact of charge trapping on the memory window (MW) of HfO_2_-based ferroelectric field effect transistors (FeFETs) will permit the design of program and erase protocols, which will guide the application of these devices and maximize their useful life. The effects of charge trapping have been studied by changing the parameters of the applied program and erase pulses in a test sequence. With increasing the pulse amplitude and pulse width, the MW increases first and then decreases, a result attributed to the competition between charge trapping (CT) and ferroelectric switching (FS). This interaction between CT and FS is analyzed in detail using a single-pulse technique. In addition, the experimental data show that the conductance modulation characteristics are affected by the CT in the analog synaptic behavior of the FeFET. Finally, a theoretical investigation is performed in Sentaurus TCAD, providing a plausible explanation of the CT effect on the memory characteristics of the FeFET. This work is helpful to the study of the endurance fatigue process caused by the CT effect and to optimizing the analog synaptic behavior of the FeFET.

## 1. Introduction

Ferroelectric field effect transistors (FeFETs) have become one of the most promising candidate devices for emerging nonvolatile memory applications due to the discovery of the ferroelectricity in HfO_2_ thin films [1,2,3]. HfO_2_-based FeFETs have the advantages of excellent compatibility with CMOS (Complementary Metal Oxide Semiconductor) processes, high scalability, and mature manufacturability, which are lacking with traditional perovskite ferroelectric materials [4,5,6]. Significant progress has been made in promoting the development of the advanced technology, improving device performance, and exploring novel device applications for these HfO_2_-based FeFETs [7,8,9,10]. For example, they were integrated into technology nodes below 28 nm by fabricating FeFETs in non-planar configurations [11]; the performance of the FeFETs device was optimized by changing the device structure [12,13], and basic logic operation was realized [14]. These devices are also increasingly drawing the attention of the emerging neuromorphic and analog-in-memory computing sectors, showing the great prospects for applications of this ferroelectric technology [15].

However, charge trapping (CT) in HfO_2_-based FeFETs is a major challenge, as it limits their full application. HfO_2_ is a dielectric material with high density intrinsic defects. The HfO_2_/interlayer and interlayer/semiconductor interfaces of HfO_2_-based FeFETs typically have defects, which will trap electrons and holes [16,17,18]. The threshold voltage (*V*_T_) shift caused by CT is opposite to the *V*_T_ shift caused by ferroelectric switching (FS), so that the memory window (MW) of the device is reduced. With an increase in the number of cycles, the newly generated defects caused by the cycles capture more charges until the MW disappears [15]; the endurance of the conventional HfO_2_-based FeFET is limited to 10^4^–10^5^ program (PGM) and erase (ERS) cycles, which greatly limits the application of the FeFET in many fields [19,20]. Therefore, understanding the interaction between CT and FS is essential for the optimization of FeFETs. Currently, some progress has been made in the study of the CT effect in FeFET devices [21,22,23], but no convincing conclusion has been reached.

This work uses an FeFET based on Hf_0.5_Zr_0.5_O_2_ (HZO), and systematically studies the CT effect in a fabricated W/TiN/HZO/SiO_2_/Si gate stacked FeFET by applying pulse sequences with various controlled pulse amplitudes and widths to the gate of the device, and with various read delay times. The competition between CT and FS is analyzed by a single-pulse technique. The pulse width and pulse amplitude testing schemes are used to study the influence of the CT effect on the conductance modulation characteristics of the FeFET. Finally, the mechanism of the CT effect on the MW of the FeFET is analyzed using the Sentaurus TCAD tool.

The rest of this paper is arranged as follows. Section 2 demonstrates the fabrication and testing methods of the FeFET; Section 3 introduces the effects of program/erase pulse amplitude, pulse width and read delay time on the MW of the FeFET, the competitive relationship between the CT and FS, the influence of the CT effect on the conductance modulation characteristics, and the *P-V* characteristics are tested to verify the ferroelectric performance of FeFET, and the endurance is also tested to confirm the influence of CT effect on FeFET; in Section 4, the mechanism of the CT effect on the MW is analyzed by using TCAD tool. Section 5 provides the conclusion.

## 2. Devices and Methods

**Figure 1:**
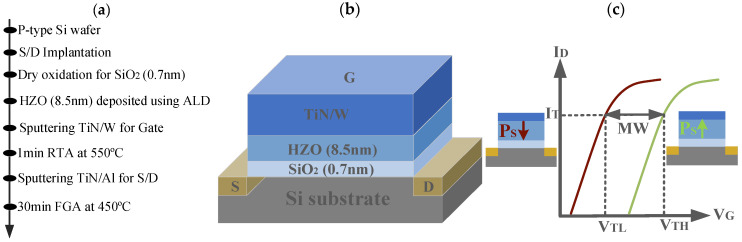
(**a**) Key fabrication process steps and (**b**) schematic structure for the fabricated Hf_0.5_Zr_0.5_O_2_ (HZO) ferroelectric field effect transistor (FeFET) devices. (**c**) The transfer characteristic curves (*I*_D_-*V*_G_) under the opposite spontaneous polarization (*P*_S_) directions for the FeFET.

An n-channel FeFET with W/TiN/HZO/SiO_2_/Si gate stacks is fabricated on 8-inch p-Si (100) substrates using a gate-last process. Figure 1a,b show the key process steps and structure for the fabricated devices. The fabrication starts from the p-type Si substrate, and the Source (S) and Drain (D) are formed by implanting As ions. After that, an SiO_2_ insulator layer 0.7 nm thick is grown by oxidation in the O_3_ atmosphere. An HZO film 8.5 nm thick is formed by atomic layer deposition (ALD). The SiO_2_ layer and the HZO layer constitute the gate dielectric films. Then, TiN/W and TiN/Al are, respectively, deposited by sputtering as the gate metal and S and D metal. The HZO film is crystallized by rapid thermal annealing (RTA) in an N_2_ atmosphere at 550 °C for 60 s. Lastly, forming gas annealing (FGA) is performed.

The electrical characteristics of the FeFET device are measured using Agilent B1500A semiconductor device analyzer with SMUs and WGFMUs for DC and pulsed electrical measurements, respectively [24]. The gate width (*W*) and length (*L*) of the device used for MW tests are 150 μm and 10 μm, respectively. The memory effect in the FeFET depends on the polarization reversal in the gate stack. The polarization is downward or upward at a sufficiently large positive or negative gate voltage, respectively, corresponding to a low threshold voltage (*V*_TL_) state and a high threshold voltage (*V*_TH_) state [25]. The relationship between the transfer characteristic curves (*I*_D_-*V*_G_) and the polarization direction is shown in Figure 1c. The write operation is performed by applying PGM and ERS pulses, and the read operation is performed in a static scanning mode.

The gate stack consisting of a 10 nm thick TiN top gate electrode, an 8.5 nm thick HZO layer, and a 0.7 nm thick SiO_2_ layer on the substrate is confirmed by transmission electron microscopic (TEM), as shown in Figure 1c. It can be observed that the HZO layer is polycrystalline, indicating the multidomain structure in the HZO-based FeFETs. Moreover, the multilayer gate stack shows sharp interfaces, indicating that the high-quality film of FeFET device.

**Figure 2:**
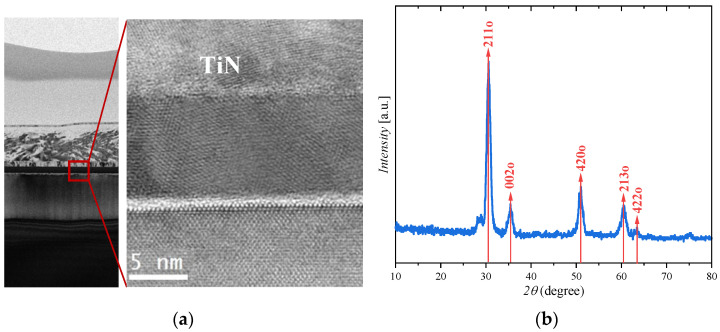
(**a**) Cross-sectional transmission electron microscope (TEM) image of the FeFET with gate stack of TiN/HZO/SiO_2_ on Si substrate. (**b**) The X-ray diffraction (XRD) pattern of the HZO thin films.

The crystal structures of the HZO films are also examined. Figure 2b presents X-ray diffraction (XRD) pictures of the FeFET. The measured peaks are well matched with the orthorhombic phase (PDF#83–0808) of the ferroelectric films. The XRD peaks are observed at the 2θ values of the superimposed orthogonal, indicating the ferroelectric properties of the prepared HfO_2_-based FeFET.

**Figure 3:**
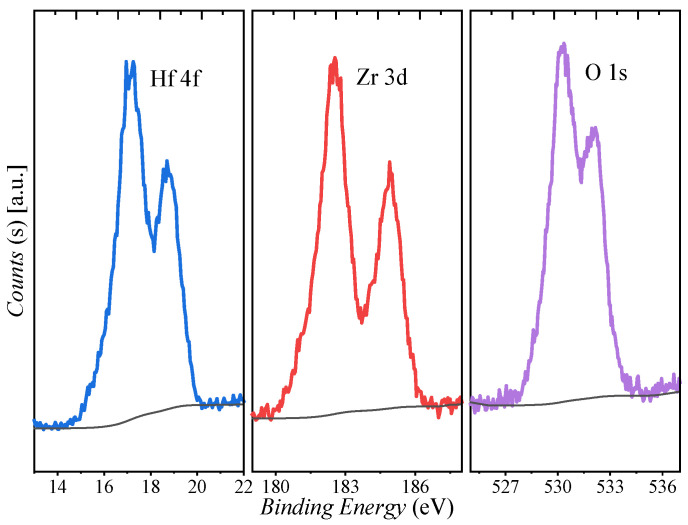
The XPS survey scan of the deposited 8.5 nm HZO layer.

The X-ray photoelectron spectroscopy (XPS) of the HZO thin films is shown in Figure 3. The characteristic peaks of Hf 4f, Zr 3d, and O 1s are observed in the general survey spectra. Two components are identified at 16.9 and 18.6 eV corresponding to the hafnium oxide O–Hf–O bonds. Moreover, the Hf 4f doublet spin-orbit splitting and the peak intensity ratio are 1.7 eV and ∼0.73, respectively, in good agreement with the reported values [26]. Similarly, the Zr 3d spectrum consists of two spin-orbit splitting peaks at 182.5 and 184.9 eV. The energy splitting of 2.4 eV and the peak intensity ratio of ∼0.75 are consistent with Zr 3d found in the literature [27]. The ratio of the atomic percentage of Hf and Zr is about 1.05:1, indicating the Hf_0.5_Zr_0.5_O_2_ composition of the sample.

## 3. Experimental Results 

### 3.1. Memory Window

**Figure 4:**
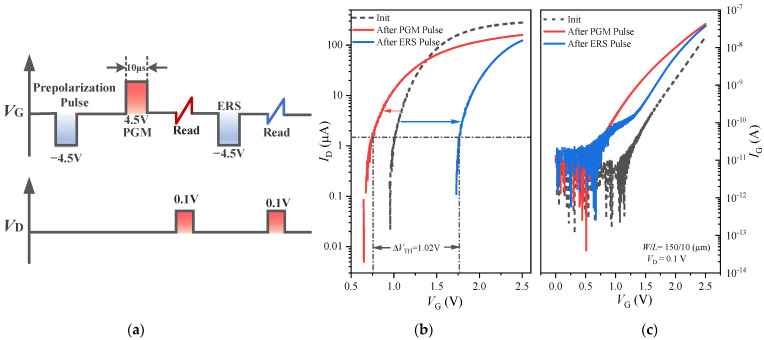
(**a**) Pulse sequence used for finding the MW of the FeFET. (**b**) *I*_D_-*V*_G_ and (**c**) *I*_G_-*V*_G_ curves of FeFET after PGM pulse and ERS pulse compared with the initial state.

Standard memory characteristics of the FeFET are measured using a pulse sequence as shown in Figure 4a: a pre-polarization pulse, a PGM pulse, an *I*_D_-*V*_G_ read, an ERS pulse, and another *I*_D_-*V*_G_ read [28]. In the figure, the amplitude of the PGM pulse is +4.5 V, and of the ERS pulse, −4.5 V; the pulse widths are 10 μs. The read *I*_D_-*V*_G_ is measured from −1 V to 2.5 V taking 100 µs, and the drain voltage (*V*_D_) is set to *V*_D_ = 100 mV. The *I*_D_-*V*_G_ curves under the three conditions of no pulse (initial state), positive PGM pulse, and negative ERS pulse are tested and shown in Figure 4b. For the n-type FeFET, compared with the *I*_D_-*V*_G_ curve in the initial state, the negative ERS pulse causes the *I*_D_-*V*_G_ curve to shift to the right and the *V*_T_ to increase, while the positive PGM pulse causes the *I*_D_-*V*_G_ curve to shift to the left and the *V*_T_ to decrease. This is because the polarization state of the ferroelectric film in the gate stack changes when the gate is applied with the PGM pulse and ERS pulse, resulting in a change in the threshold voltage of the FeFET. The MW value of the FeFET is obtained by extracting the threshold voltage difference (Δ*V*_T_) after the PGM and ERS pulses from the *I*_D_-*V*_G_ curves. In this work, we define *V*_T_ as the particular *V*_G_ at which the *I*_D_ = 10^−7^ × *W*/*L* A [29]. Figure 4b shows that the MW of the FeFET is 1.02 V (MW = *V*_TH_ − *V*_TL_). This shows that the fabricated FeFET device has good storage characteristics.

The *I*_G_-*V*_G_ curves in Figure 4c show that, after applying the PGM and ERS pulse, the IG is larger than that in the initial state. The traps generated under the PGM and ERS pulses will trap electrons, and some electrons may enter the gate through the gate dielectric layer to form the gate leakage current, thus causing the IG increase after the PGM and ERS pulses, as compared with the initial state.

#### 3.1.1. Memory Window under Various PGM and ERS Pulse Amplitudes

**Figure 5:**
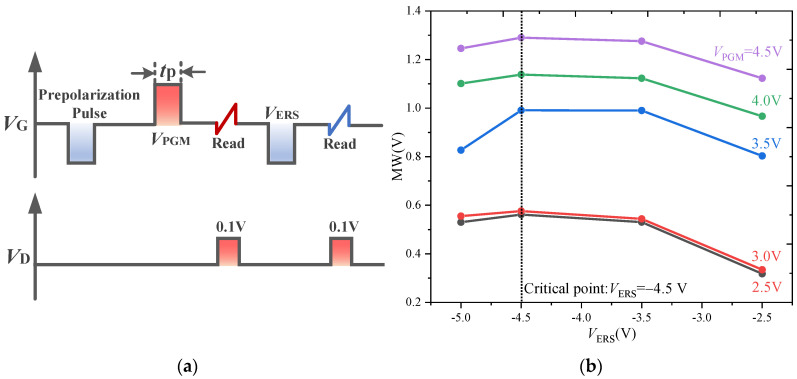
(**a**) Pulse sequence used for MW test at various *V*_PGM_ of the FeFET. (**b**) Variation of MW with respect to *V*_ERS_ for various values of *V*_PGM_ in FeFET.

The influence of the CT on the MW is studied by changing the amplitudes of the PGM and ERS pulses (*V*_PGM_ and *V*_ERS_); the pulse sequence is shown in Figure 5a: the pulse width (*t*_p_) is 10 μs. The MW is plotted as functions of *V*_ERS_ for the various values of *V*_PGM_, as shown in Figure 5b.

Figure 5b shows that, under a given *V*_PGM_, the MW increases with the increase in |*V*_ERS_|, but when |*V*_ERS_| is greater than 4.5 V the MW decreases instead. For this result, the explanation is that the CT effect and FS effect act together on the FeFET, but their contributions to the MW are opposite. *V*_ERS_ = −4.5 V is the critical point. When |*V*_ERS_| is less than 4.5 V, the polarization of the ferroelectric layer increases with the increase in |*V*_ERS_| until it reaches the saturation state. In this process, the FS as the dominant effect causes the MW to gradually increase. Although |*V*_ERS_| higher than 4.5 V will lead to further switching of the polarization, the increased CT effect in this voltage range overcompensates the influence of the ferroelectric polarization on the FeFET, resulting in a decrease in MW [28]. The relationship between CT and FS will be described in Section 3.2.

#### 3.1.2. Memory Window under Various PGM and ERS Pulse Widths

The influence of the CT effect on the MW is studied by changing the width of the PGM and ERS pulses (*t*_P_). The pulse sequence is shown in Figure 5a. The pulse amplitude (*V*_PGM_/*V*_ERS_) is ±4.5 V.

**Figure 6:**
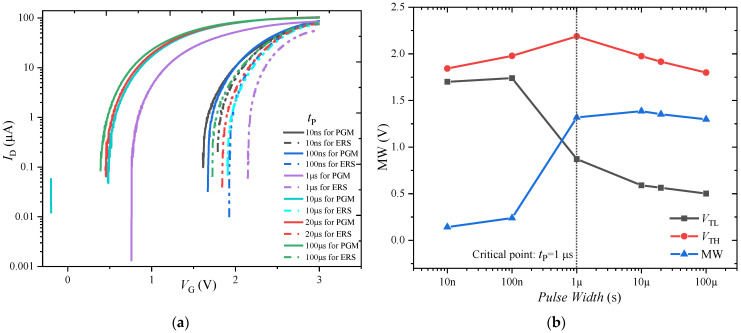
(**a**) *I*_D_-*V*_G_ characteristics of FeFET for varying PGM and ERS pulse widths. (**b**) Variation of *V*_TL_, *V*_TH_, and MW with respect to various values of pulse width in FeFET.

Figure 6a shows that the ID-VG curves under the various PGM pulse width values change greatly, and especially that *t*_P_ = 1 μs is the critical point. When *t*_P_ > 1 μs, the ID-VG curves under the PGM pulse shift significantly to the left. The variation of low threshold voltage (VTL), high threshold voltage (VTH) and MW value with respect to the pulse width are extracted according to the ID-VG curves, as shown in Figure 6b. When *t*_P_ = 10 μs, the MW value reaches the maximum. The above phenomena can be explained by noting that in the PGM and ERS pulse sequence, the CT and FS act on the FeFET simultaneously, but compete. With the increase in the pulse width, the polarization will gradually completely reverse to increase the MW, and the probability of traps near the interface will also increase to weaken the MW. When *t*_P_ < 1 μs, the short pulse duration limits the polarization switching process. With the increase in the pulse width, the polarization is gradually completely reversed, and the increase in the FS on the MW is stronger than the weakening of the CT on the MW, resulting in the increase in the MW with the increase in the pulse width. When *t*_P_ > 10 μs, the polarization has reached the limit state of complete inversion, and the larger pulse width leads to more charge capture. The decrease in the MW due to the CT effect is stronger than the increase in the MW due to the FS effect, resulting in a slight reduction in the MW with the increase in the pulse width.

#### 3.1.3. Memory Window under Various Read Delay Times

**Figure 7:**
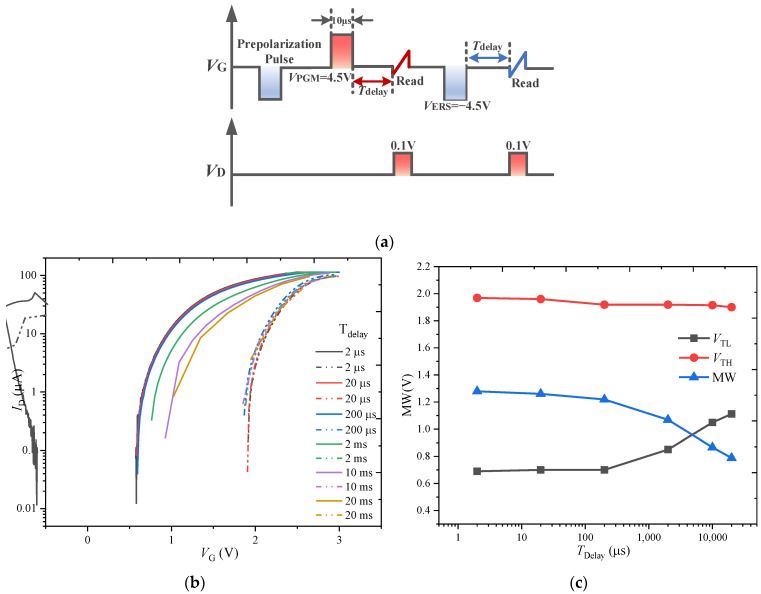
(**a**) Pulse sequence used for MW test at various *T*_delay_ of the FeFET. (**b**) *I*_D_-*V*_G_ characteristics of FeFET for varying *T*_delay_. (**c**) Variation of *V*_TL_, *V*_TH_, and MW at various values of *T*_delay_ in FeFET.

The time from the end of the write operation to the start of the read operation for the FeFET is defined as the read delay time (*T*_delay_). The effect of the read delay time on the MW is studied by applying the pulse sequence shown in Figure 7a. While the write pulses are fixed to ±4.5 V and 10 µs to ensure the complete switching of the polarization, the *T*_delay_ is varied from 2 μs to 20 ms.

The *I*_D_-*V*_G_ curves for various *T*_delay_ are shown in Figure 7b. With the increase in *T*_delay_, the *I*_D_-*V*_G_ curve after the PGM operation obviously shifts to the right, resulting in the reduction in the MW. Figure 7c shows the relationship between the *V*_TL_, *V*_TH_, and MW with *T*_delay_: *V*_TH_ has no obvious change with the *T*_delay_, while *V*_TL_ shows some retention degradation for *T*_delay_ > 200 μs. The above phenomena can be explained by noting that there is reverse switching of ferroelectric domains under the depolarization field [20]; the number of reverse-switching domains increases with the increase in *T*_delay_, resulting in the reduction in the MW. However, the significant difference between the *I*_D_-*V*_G_ curves under the PGM and ERS pulses with the increase in *T*_delay_ indicates that the depolarization field is not the main reason for the decrease in MW. On the other hand, the traps generated under the programming and erasing pulses have more chances to capture the charge with the increase in *T*_delay_, resulting in the reduction in the MW. In addition, the difference in the change degree of *V*_TH_ and *V*_TL_ with *T*_delay_ is caused by the differing abilities to generate traps under the PGM and ERS pulses.

### 3.2. Charge Trapping and Ferroelectric Switching Effect

**Figure 8:**
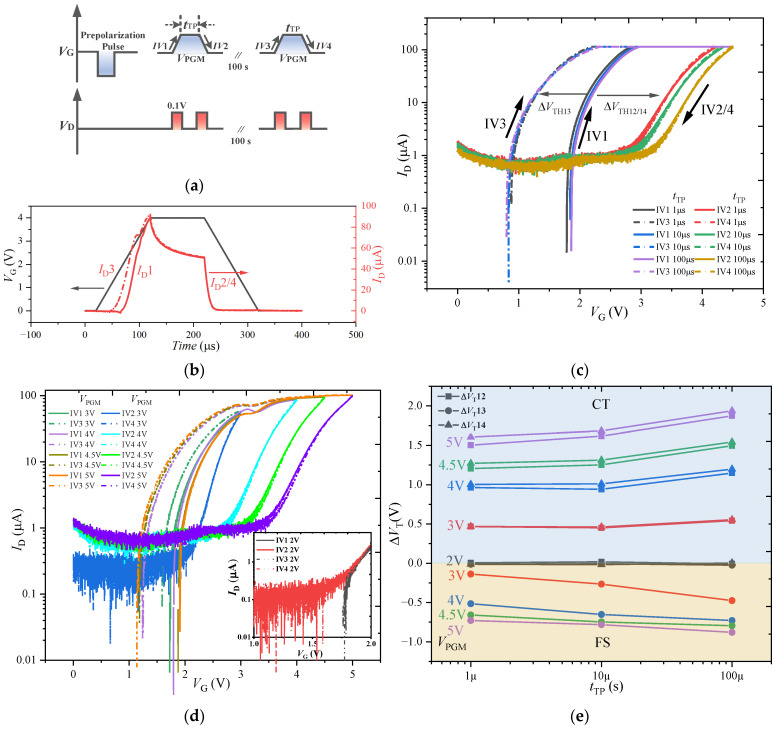
(**a**) Pulse sequence with two single pulses used for CT and FS competition test of the FeFET. (**b**) Transient current response of FeFET under two single pulses: *I*_D_1 and *I*_D_3 correspond to the rising edges *IV*1 and *IV*3, *I*_D_2 and *I*_D_4 correspond to the falling edges *IV*2 and *IV*4. *I*_D_-*V*_G_ characteristics measured on the rising (*IV*1) and falling (*IV*2) edges of the first single-pulse and the rising (*IV*3) and falling (*IV*4) edges of the second single-pulse (**c**) with various pulse widths, and (**d**) with various pulse amplitudes. (**e**) Variation of Δ*V*_T_ with respect to *t*_TP_ for various values of *V*_PGM_ under two single pulses.

The FS and CT effects occur simultaneously in the FeFET. The contributions of these two effects to the MW are opposite: Under the positive PGM pulse, the CT leads the *V*_T_ shift to the right, the FS leads the *V*_T_ shift to the left; under the negative ERS pulse, the CT leads the *V*_T_ shift to the left, the FS leads the *V*_T_ shift to the right. Therefore, the superposition of the two effects will lead to the reduction in the MW of the FeFET. The competition between the FS and CT in the FeFET is studied by using the single-pulse *I*_D_-*V*_G_ method [21]. The gate-pulse sequence shown in Figure 8a is used for this purpose. A first negative pulse (−4.5 V, 10 µs) is applied to establish a negative saturation polarization state, and two consecutive positive amplitude single pulses are applied to analyze the CT effect superimposed on the ferroelectric polarization switching. The specific test principle is as follows. During the first positive pulse, the dominant mechanism is determined by the *V*_T_ offset between the *I*_D_-*V*_G_ curves obtained on the rising edge (*IV*1) and the falling edge (*IV*2) of the pulse: if Δ*V*_T_ < 0, the FS dominates; if Δ*V*_T_ > 0, the CT dominates. After the first positive pulse, the FeFET is in a positive polarization state, and the dominant mechanism is determined according to the *V*_T_ offset between the *I*_D_-*V*_G_ curves obtained on the rising edge of the second pulse (*IV*3) and the rising edge of the first pulse (*IV*1). The purpose of 100 s interval between two single pulses is to recover the captured charge.

Figure 8b shows the corresponding current transient response when two single pulses (*V*_PGM_ = 4.5 V, *t*_TP_ = 10 µs) are applied to the gate, wherein the black solid line is the applied two single pulses (two pulses coincide), the red solid line *I*_D_1 corresponds to the rising edge *IV*1, and the red dotted line *I*_D_3 corresponds to the rising edge *IV*3. *I*_D_2 and *I*_D_4 correspond to falling edges *IV*2 and *IV*4. Figure 8b shows that *I*_D_3 is shifted to the left relative to *I*_D_1, and *I*_D_2 and *I*_D_4 are almost coincident.

The *t*_TP_ and *V*_PGM_ of the two single pulses in Figure 8a are changed to analyze the competitive relationship between the CT and FS, multiple groups of *I*_D_-*V*_G_ curves varying with *t*_TP_ and *V*_PGM_ are obtained, as shown in Figure 8c,d, respectively. For Figure 8c, *IV*2 shifts to the right relative to *IV*1 during the first positive pulse, Δ*V*_T_12 = *V*_T_2 − *V*_T_1 > 0 (*V*_T_1 and *V*_T_2 represent the threshold voltages extracted from the *IV*1 curve and the *IV*2 curve, respectively), indicating that the CT dominates the threshold voltage shift. During the second positive pulse, *IV*3 shifts to the left relative to *IV*1, Δ*V*_T_13 = *V*_T_3 − *V*_T_1 < 0, indicating that the polarization switching dominates the threshold voltage shift. In addition, Δ*V*_T_12 increases with the increase in *t*_TP_, indicating that the trapped charge increases; Δ*V*_T_13 has almost no change with the increase in *t*_TP_, which further indicates that after the first pulse is applied, the FS dominates the change of threshold voltage rather than the charge detrapping.

For Figure 8d, the *I*_D_-*V*_G_ curves have no obvious shift when the *V*_PGM_ ≤ 2 V, as shown in the inset, indicating that the CT and FS effect of the FeFET are not significant under small *V*_PGM_. *IV*2/4 and *IV*3 are significantly shifted in opposite directions relative to *IV*1 with the increase in the *V*_PGM_. This shows that the CT and FS effect exist simultaneously. However, when the *V*_PGM_ ≥ 4.5 V, the offset of *IV*3 relative to *IV*1 does not obviously increase with the increase in *V*_PGM_, while the offset of *IV*2/4 relative to *IV*1 still increases with the increase in *V*_PGM_. This indicates that the CT effect will be the dominant mechanism of the change of the MW for the FeFET when the pulse amplitude is greater than 4.5 V. The relationship between *V*_PGM_ and Δ*V*_T_ is summarized in Table 1, where the symbol “↑” in the table indicates Δ*V*_T_ increase It can also explain that in Section 3.1.1, when |*V*_ERS_| > 4.5 V, the MW value decreases instead with the increase in the pulse amplitude.

Figure 8e summarizes the variation of Δ*V*_T_ with respect to *t*_TP_ for various values of *V*_PGM_, where Δ*V*_T_12 and Δ*V*_T_14 increase with the increase in the pulse width under the various amplitudes, indicating that the amount of the trapped charge increases. In addition, |Δ*V*_T_13| increases significantly with the increase in the *t*_TP_ when the *V*_PGM_ = 3 V, indicating that the *t*_TP_ also limits the inversion of the ferroelectric domain under the small *V*_PGM_. The most important thing is that |Δ*V*_T_13| does not increase significantly when the *V*_PGM_ increases to 4.5 V, indicating that the CT effect plays a dominant role in the FeFET compared with the FS effect at large pulse amplitude.

### 3.3. Conductance Modulation Characteristic Influenced by Charge Trapping Effect

**Figure 9:**
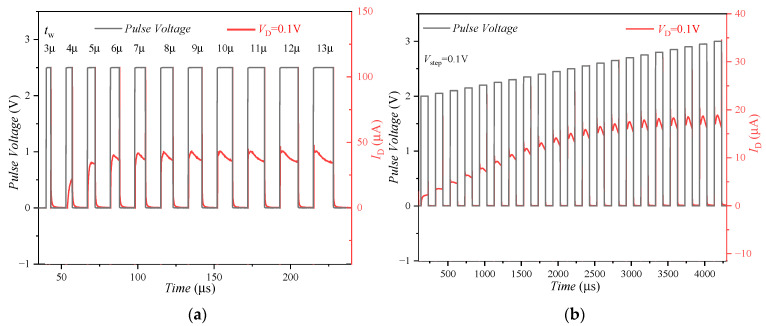
Effect of CT on (**a**) pulse width and (**b**) pulse amplitude modulation channel conductivity for FeFET. The gate of FeFET is applied with a pulse sequence with gradually increasing pulse width and pulse amplitude, and the drain current value is read out under a fixed drain voltage (*V*_D_ = 0.1 V).

The potential of the FeFET to exhibit analog synapses behavior has been investigated by the pulse width and amplitude modulation scheme in [30,31,32,33]. However, the experimental data in this work show that the channel conductance modulated by the pulse width and pulse amplitude will also be affected by the CT effect. The gate of the FeFET is applied with a pulse sequence that continuously increases the pulse width, and the drain of the FeFET is applied with a voltage of 0.1 V to read the drain current value, as shown in Figure 9a. When the pulse width is small, the FS effect dominates, and the drain current (*I*_D_) increases with the increase in the number of pulses, showing the potentiation characteristic; With the increase in the pulse width, the probability of the charge capture increases, and the CT effect increases gradually, which inhibits the increase in the *I*_D_, that is, the potentiation characteristics of the FeFET are suppressed.

Similarly, when a pulse sequence with a continuously increasing pulse amplitude is applied to the gate of the FeFET, as shown in Figure 9b, the *I*_D_ increases with the number of pulses, FS is the dominant effect, showing the potentiation characteristic. When all domains in the ferroelectric layer are reversed, the polarization in the ferroelectric layer reaches the saturation state, and the current will not increase. However, during the process of the pulse application, the *I*_D_ has a peak value, which can be explained as follows: with the increase in the pulse application time, the CT effect becomes the dominant mechanism, leading to the reduction in the *I*_D_.

### 3.4. P-V Characteristics

**Figure 10:**
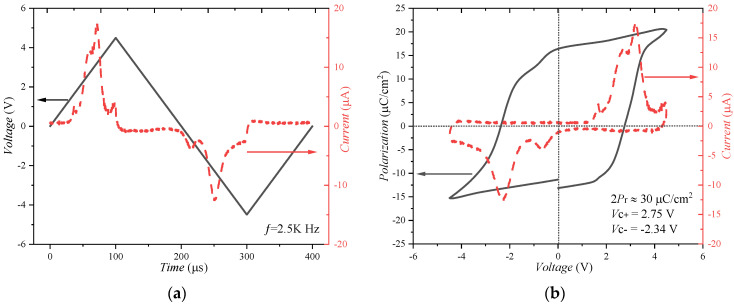
(**a**) Transient current response (red dotted line) of the FeFET under triangular excitation gate voltage (black solid line) during polarization measurements at a frequency of 2.5 kHz, and (**b**) resulting Current–Voltage (red dotted line) and Polarization–Voltage (black solid line) curve.

The *P*-*V* characteristics are measured by applying a triangular pulse sequence to the gate of the FeFET at 2.5 kHz as shown by the black solid line in Figure 10a, and short the Source, Drain, and substrate to the ground [28,34,35]. The maximum values of the triangular pulse sequence are *V*_P_ = 4.5 V and *V*_N_ = −4.5 V, respectively. The transient current response of a ferroelectric capacitor under triangular excitation gate voltage is shown by the red dotted line in Figure 10a. There are two characteristic current peaks in the current versus the time curve, corresponding to the domain switching under the coercive voltage (*V*_C_).

The polarization measurement is actually a measurement of charge in the capacitor given by Equation (1) [34,36].

(1) Q=P⋅A

where *Q* is the total charge in the ferroelectric, *A* is the capacitor area, and *P* is the polarization. Polarization can thus be obtained by the integration of the measured switching current (*I*) with respect to time, as shown in Equation (2) [34,36].

(2) $$P=\frac{\int Idt}{A}$$

The Polarization–Voltage (*P*-*V*) curve and Current–Voltage (*I*-*V*) are shown in Figure 10b. The *P*-*V* curve shows a typical polarization hysteresis loop with a dual remanent polarization (2*P*_r_) value of 30 μC/cm^2^, and a positive and negative coercive voltage of 2.75 V and −2.34 V, respectively. The clear polarization switching behavior can be observed in the *P*-*V* curve. The Current–Voltage curve shows a single loop hysteresis with two consistent peaks, which is also a typical characteristic of ferroelectric materials [28].

### 3.5. Endurance

**Figure 11:**
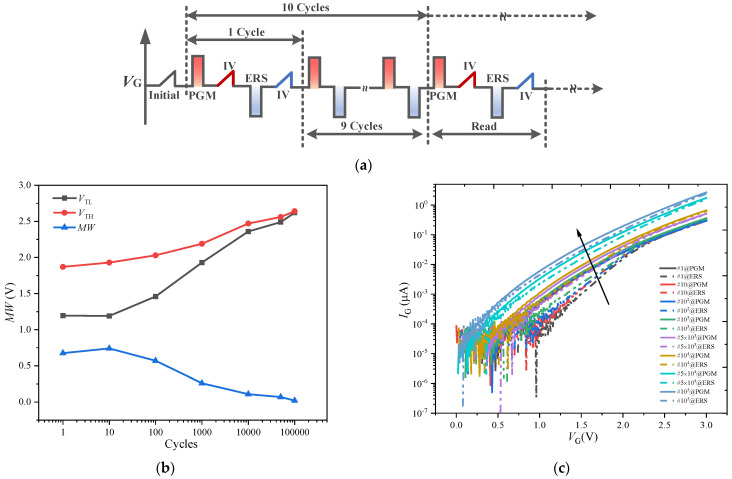
(**a**) Pulse sequence used for endurance measurements of the FeFET. (**b**) Variation of MW, *V*_TH_, and *V*_TL_ with respect to cycles number. *V*_TH_ and *V*_TL_ increase with the increase in endurance cycles, the MW disappears at 10^5^ cycles. (**c**) *I*_G_-*V*_G_ curves under various endurance cycles, where the black arrow indicates that the gate current increases with the number of endurance cycles.

The endurance of the FeFET is tested by applying a pulse sequence with a *V*_PGM_ = 4 V, *V*_ERS_ = −4 V and *t*_p_ = 1 μs to the gate as shown in Figure 11a. The *I*_D_-*V*_G_ curve is tested after applying the PGM and ERS pulses, and the threshold voltage is extracted from the *I*_D_-*V*_G_ curve. Figure 11b shows the degradation relationship of the MW with the number of cycles. Both the *V*_TH_ and *V*_TL_ increase with the number of cycles, which is attributed to the CT effect [22]. When the number of cycles reaches 10^5^, the MW almost disappears.

Figure 11c shows the relationship between the gate current and the gate voltage (*I*_G_-*V*_G_) under various cycles. When the number of cycles reaches 10^4^, the gate current increases significantly. When the number of cycles reaches 10^5^, the *I*_G_ increases by an order of magnitude compared with the initial state.

## 4. Discussion

**Figure 12:**
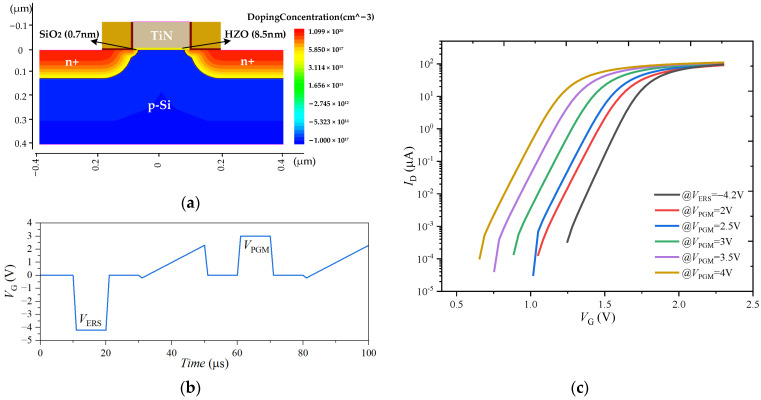
(**a**) Simulated structure of FeFET in Sentaurus TCAD. (**b**) Program/Erase pulse scheme along with the read cycle (sweep from −0.2 V to 2.5 V) for FeFET simulation. (**c**) Simulated *I*_D_-*V*_G_ characteristics of FeFET for varying *V*_PGM_.

To provide a plausible explanation of the CT behavior in the FeFET, a theoretical investigation is performed in Sentaurus TCAD. The FeFET structure, as shown in Figure 12a, is built with the p-type Si substrate, a 0.7 nm SiO_2_ interlayer, an HZO ferroelectric 8.5 nm thick, and TiN metal. The ferroelectric is described with the dynamic Preisach model [37], and the model parameters are consistent with the measured experimental data of the FeFET, *P*r is 15 μC/cm^2^, *P*s is 20 μC/cm^2^, and *E*c is 2.5 MV/cm. Acceptor-type traps (Trap density = 1 × 10^13^ cm^−3^) are injected at the channel/interface layer of the FeFET for the TCAD simulation [38]. The pulse scheme shown in Figure 12b is used for the simulation of the FeFET MW, and the amplitude of the programming pulse is changed to evaluate the impact of the CT on the MW.

Under a fixed erasing pulse amplitude (*V*_ERS_ = 4.2 V), the MW increases with the increase in the amplitude of the *V*_PGM_ pulse, as shown in Figure 12c. This is because with the increase in the PGM amplitude, the polarization intensity in the ferroelectric layer gradually increases, resulting in the shift of the *I*_D_-*V*_G_ curves’ decrease in the threshold voltage. In this process, the FS effect is the dominant mechanism, which is consistent with the test results of the FeFET.

**Figure 13:**
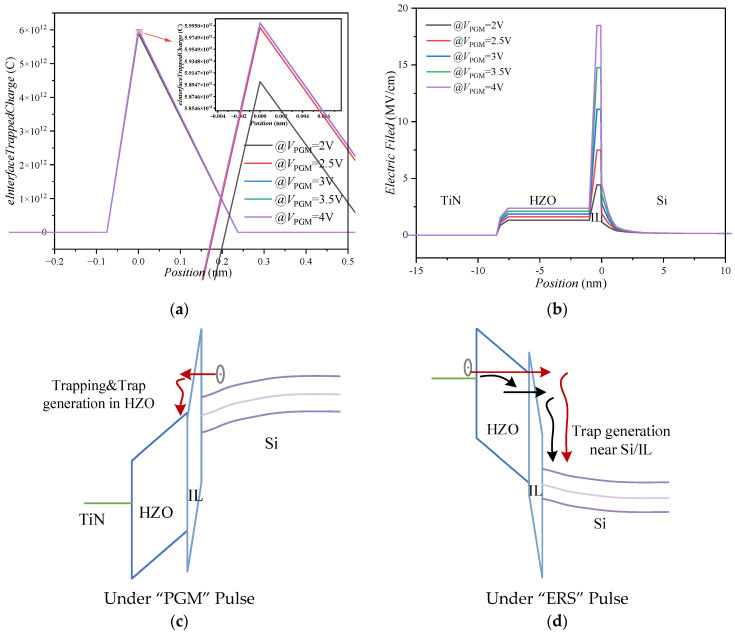
(**a**) Interface trapped charge varies with the voltage of program pulse. (**b**) Electrical field and conduction band diagram in FeFET using TCAD tools at the various voltages of the program pulse. Schematic diagram of energy band under (**c**) PGM and (**d**) ERS pulses, where the red and black arrows indicate that electrons flow through HZO through FN tunneling and trap-assisted conduction, respectively.

The CT effect in the process of increasing the amplitude for the PGM pulse is further analyzed. When the amplitude of the *V*_PGM_ increases from 2 V to 2.5 V, the number of charges trapped by the interface traps increases significantly, as shown in the inserted figure in Figure 13a, which counteracts the ability of the FS effect to increase the MW, resulting in a slightly smaller increase in the MW. When the *V*_PGM_ is greater than 2.5 V, the number of trapped charges in the interface still increases, but the FS effect, as the dominant mechanism, makes the MW significantly increased. From the simulation results and experimental data, it can be seen that the CT effect strongly depends on the pulse conditions applied by the gate. In order to reduce the impact of the CT effect on the FeFET MW and increase the endurance of the device, appropriate programming and erasing conditions can be designed.

The variations of electric field strength in the ferroelectric layer and interface layer with the amplitude of *V*_PGM_ are shown in Figure 13b. The interlayer electric field is strengthened by the polarization pointing at the channel under the PGM pulse. It will facilitate the electron injection into the gate stack.

Figure 13c,d show the energy band diagram under the PGM and ERS pulse train, respectively. During the PGM pulse, electrons tunnel through the interface layer (IL) and enter the HZO region. Energy will be lost in this process, which will generate border traps near the HZO/IL interface [22]. However, some electrons may be trapped; during the ERS pulse, electrons flow through HZO by Fowler–Nordheim (FN) or by trap-assisted conduction and remain “hot” when entering the Si interface. Some of them may generate interface traps while losing energy. Traps generated under the PGM and ERS pulse will capture charges. The number of traps generated and charges captured is related to the amplitude and width of the PGM and ERS pulse, which compete with the FS effect and affect the memory window of the FeFET.

## 5. Conclusions

The effect of CT on the performance of W/TiN/HZO/SiO_2_/Si gate stacked FeFET devices is studied. The competition between CT and FS is analyzed by a single-pulse technique. When the amplitude of the single-pulse is increased to 4.5 V, the contribution of CT is stronger than that of FS for the FeFET, and the MW is reduced. Due to the CT effect, the endurance cycle of the FeFET is only 10^5^. In addition, in the analog synaptic behavior of the FeFET, the conductance modulation characteristics are also affected by the CT effect, and the continuous increase in conductance is inhibited. The simulation results of Sentaurus TCAD explain the mechanism of the CT effect on the MW of the FeFET. This work lays a foundation for the study of alleviating the endurance fatigue process caused by the CT effect and enhancing the analog synaptic behavior of the FeFET.

## Figures and Tables

**Table 1:** Variation of Δ*V*_T_ at various *V*_PGM_ by the single-pulse *I*_D_-*V*_G_ method.

*V*_PGM_	Δ*V*_T_12	Δ*V*_T_13	Dominant Role
≤2 V	-	-	-
>2 V and ≤4.5 V	↑	↑	CT and FS
>4.5 V	-	↑	CT

## Data Availability

Not applicable.
